# The bacterial diversity and potential pathogenic risks of giant panda-infesting ticks

**DOI:** 10.1128/spectrum.02197-24

**Published:** 2025-06-10

**Authors:** Rui Ma, Yi Shi, Wei Wu, Chong Huang, Fei Xue, Rong Hou, Yanshan Zhou, Jiang Gu, Feifei Feng, Xiang Yu, Jiabin Liu, Zusheng Li, Long Zhang, Guanwei Lan, Chao Chen, Wenlei Bi, Xinqiang Song, Mingxia Fu, Hong Yang, Haijun Gu, Jiandong Yang, Dunwu Qi

**Affiliations:** 1 Sichuan Key Laboratory of Conservation Biology for Endangered Wildlife, Chengdu Research Base of Giant Panda Breeding 635499, Chengdu, Sichuan, China; 2 College of Animal Science and Technology, Sichuan Agricultural University 719503 , Chengdu, Sichuan, China; 3 Daxiangling Nature Reserve, Yaan, Sichuan, China; 4 Sichuan Forestry and Grassland Bureau, Chengdu, China; Lerner Research Institute, Cleveland, Ohio, USA

**Keywords:** ticks, *Ixodes ovatus*, microbial community, tick-borne pathogen, metagenomics

## Abstract

As common parasites in the wild, ticks significantly limit the population growth of wild giant pandas and hinder the process of reintroducing captive giant pandas into their natural habitats. Research on microbial communities and pathogens in ticks infesting giant pandas is limited, emphasizing the need for a comprehensive investigation. To thoroughly investigate the microbial communities in giant panda-infesting ticks, particularly potential pathogens, we analyzed 246 ticks collected from the ears of wild-living giant pandas using 16S rRNA and metagenomic sequencing. We found that the microbial diversity in female ticks was significantly enriched in summer. The microbial community structure carried by ticks is more significantly influenced by seasonal changes than by sex. Metagenomic results indicated that giant pandas have a higher risk of *Coxiella burnetii* infection in summer and a higher risk of *Anaplasma phagocytophilum*, *Klebsiella pneumoniae*, *Pseudomonas aeruginosa,* and *Rickettsia amblyommatis* infections in autumn. Over 90% of the ticks carried pathogens, with 82.54% harboring a single potentially pathogenic symbiont and the remaining 17.46% carrying multiple pathogens, all involving *Coxiella burnetii*. Using the CARD database, we identified a total of 121 antibiotic resistance genes (ARGs), with 76% exhibiting antibiotic efflux mechanisms. Based on the significantly associated ARGs, we provided antibiotic treatment recommendations for infections potentially caused by pathogenic symbionts. This study provides a clear answer to the potential microbial pathogen risks of ticks infesting giant pandas and offers a framework for tick-borne diseases in reintroduced wild panda populations.

**IMPORTANCE:**

Importance: The emergence of tick-borne bacterial diseases poses a serious threat to the population health of wild-living giant pandas. Ticks are obligate hematophagous ectoparasites that survive by feeding on the blood of various animal hosts and spreading pathogens. Although some previous studies have confirmed that wild ticks carried various viruses, the role of wild giant panda-infesting ticks in the bacterial community remains unknown. Here, the identification of the microbial community and antibiotic resistome in giant panda-infesting ticks revealed that most *Ixodes ovatus* ticks are potentially pathogenic symbionts, including *Anaplasma phagocytophilum*, *Coxiella burnetii*, and *Rickettsia amblyommatis*. Tick-borne disease control also needs to take into account the effects of season, sex, and antibiotic efflux resistance genes. Our findings highlight the contribution of the scientific management of tick-borne diseases in the giant panda population.

## INTRODUCTION

Ticks are specialized blood-sucking arthropods with a wide global distribution (1). Ticks are the second-most important disease vector after mosquitoes, and most species change hosts during their life cycle (2). Ticks can transmit a diverse array of pathogens to humans, livestock, pets, and wildlife, including bacteria, viruses, and parasites (3–6). Wildlife is considered a natural host and an important transmission route for ticks, with their populations potentially affected by ticks (7, 8). Smaller populations of charismatic species are a direct product of human actions and are more fragile when considering infectious diseases (9–11). As each individual becomes more valuable within a population, the loss of any individual is felt more acutely. Therefore, it is imperative to understand the disease risks faced by endangered species.

The giant panda (*Ailuropoda melanoleuca*) is globally recognized as one of the flagship and umbrella species for biodiversity conservation. In the wild, giant pandas have a 100% chance of being parasitized by ticks, which can lead to dermatitis, anemia, inflammation, and even death (12, 13). To date, a total of 35 parasite species have been identified from giant pandas. Among them, 13 are hard ticks (*Ixodidae* sp.), specifically comprising nine *Haemaphysalis* sp., three *Ixodes* sp., and one *Dermacentor* sp. (12).

It has been evidenced that *Babesia* spp. within *I. ovatus* can induce an acute decline in appetite, accompanied by lethargy and anemia in giant pandas (14). Moreover, it can also result in the reduction of red blood cell counts, erythrocyte ratios, and hemoglobin levels (14). Our previous virologic study on parasitic ticks of giant pandas revealed the presence of *Neobunyaviruses* and other viruses that potentially threaten their health (15). Studies have indicated that the pathogenic bacteria borne by *I. ovatus*, including *Borrelia burgdorferi*, *Coxiella burnetii*, and *Rickettsia* sp., are capable of spreading among multiple species (16–19). However, until now, our understanding regarding the types of pathogenic bacteria carried by *I. ovatus* on giant pandas remains completely blank.

Due to the presence of sex and seasonal differences in the microbial composition of ticks, the prevention and control of tick-borne diseases present formidable challenges (20–25). The microbial diversity of female *Ixodes scapularis* was found to be significantly lower than that of males (22). Besides, the relative abundance of tick microbial communities differed between months, with the bacterial community in the summer months (June and July) forming one cluster and the rest of the months forming another (24). In addition, the tick microbial community structure may vary depending on the presence of pathogens (26–28). Therefore, a detailed understanding of the microbial community diversity, composition, and its seasonal variation of giant panda-infesting *I. ovatus* is essential for controlling tick-borne diseases.

To evaluate the diversity and community structure of the microbiome in ticks from wild-living giant pandas and to explore the pathogenic bacteria they carry, this study utilized 16S rRNA and metagenomic sequencing. Our study aims to analyze the microbial community structure of ticks, identify the pathogens, evaluate their infectivity and abundance, and explore the metabolic pathways and antibiotic resistance genes (ARGs). The results will provide a theoretical basis for the effective prevention and control of tick-borne bacterial infectious diseases in giant pandas and to offer scientific insights for global wildlife health management.

## MATERIALS AND METHODS

### Sample collection and transportation

In June and October 2022, a total of 246 ticks were collected from three female adult giant pandas (aged 6, *n* = 2; aged 8, *n* = 1) living in Daxiangling Reintroduction Base (29°33'55.0760N—29°32'50.4740N, 102°50'13.8660E—102°51'3.1890E), the release experimental base of Chengdu Research Base of Giant Panda Breeding for captive giant pandas in Sichuan Province, China. Regular physical examinations confirmed that all pandas were in good health; no antibiotics were used in the 3 months preceding the sample collection. During the daily physical examination, specific staff members wore disposable sterile masks and disposable latex gloves to collect samples from the pandas’ ears. All the tick samples were sexed and screened for intact, undamaged adults, which were then individually dispensed into 2-mL sampling tubes and labeled. After collection, the samples were transported back to the laboratory with dry ice and stored in a −80°C ultra-low temperature refrigerator. For the experiment, 210 ticks were randomly selected for the 16S rRNA test and 36 ticks for the metagenomic test. The detailed sampling information and grouping situation are shown in Table 1.

**TABLE 1:** Sampling information and grouping situation of ticks

Group name	No. of ticks	Sex	Collection date	Sequencing method
M1	54	Male	Jul 2022	16S rRNA test
M2	14	Male	Oct 2022	16S rRNA test
F1	112	Female	Jul 2022	16S rRNA test
F2	30	Female	Oct 2022	16S rRNA test
MJ	9	Male	Jul 2022	Metagenomic test
MO	9	Male	Oct 2022	Metagenomic test
FJ	9	Female	Jul 2022	Metagenomic test
FO	9	Female	Oct 2022	Metagenomic test

### Tick species identification

**Fig 1:**
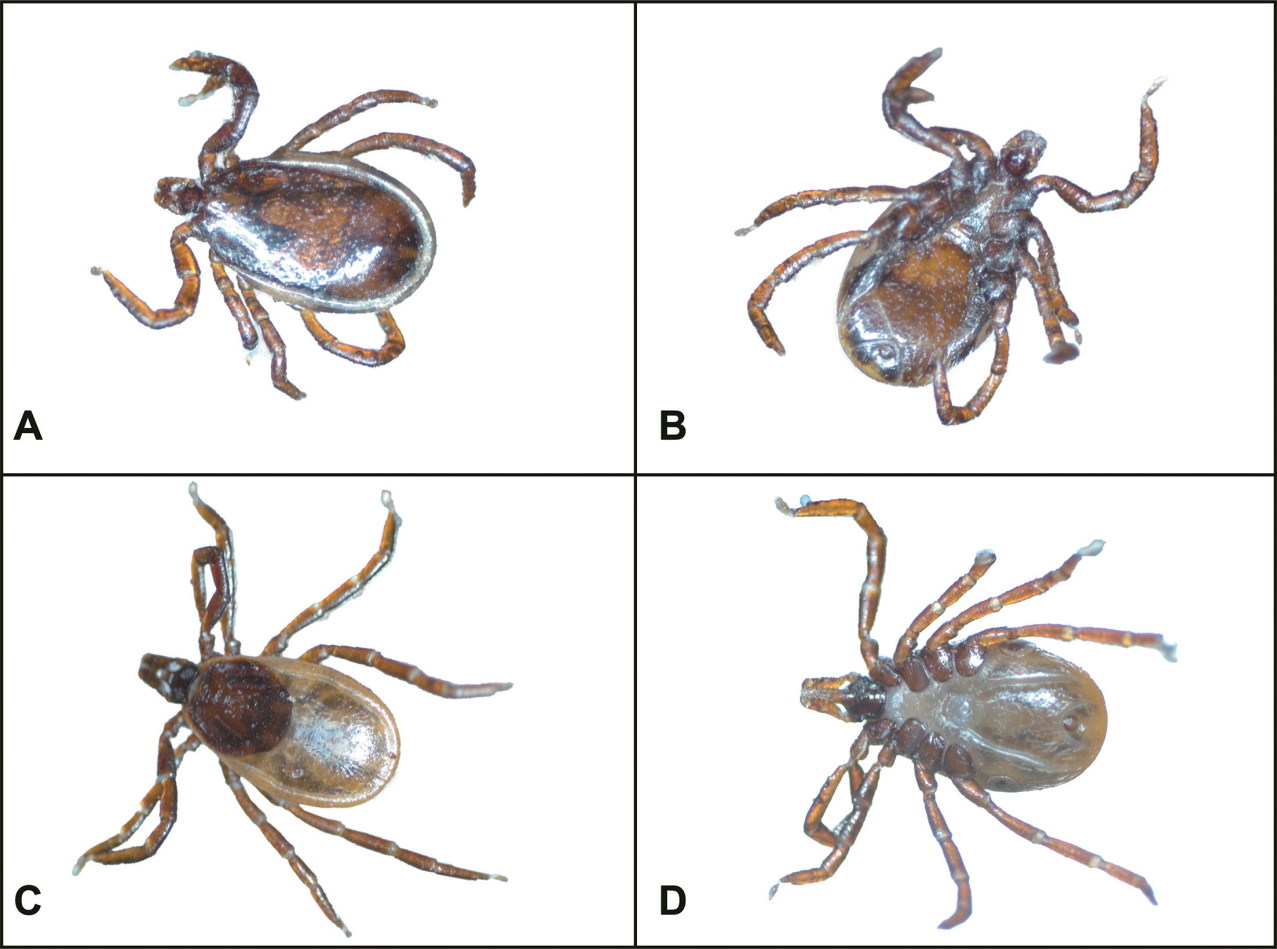
*Ixodes ovatus* collected from the giant panda. (A) Dorsal view of the adult male *I. ovatus*; (B) ventral view of the adult male *I. ovatus*; (C) dorsal view of the adult female *I. ovatus*; (D) ventral view of the adult female *I. ovatus*.

Our previous work had encompassed the collection and identification of 1,426 ticks within the identical study area (25). The identification was conducted by employing the morphological method (29) in combination with the Cytochrome Oxidase Subunit I (COI) and 16S rRNA with Basic Local Alignment Search Tool (BLAST) using The National Center for Biotechnology Information (NCBI) (30). The outcomes of this identification consistently demonstrated that all the 1,426 ticks were *Ixodes ovatus* (25). Based on that, the 246 ticks collected in the current study were subsequently identified as *I. ovatus* through morphological means (Fig. 1).

### Sample processing and DNA extraction

Considering the disturbance of microorganisms on the body surface of ticks, bleach was utilized to remove bacteria from the body surface of ticks without affecting the internal bacterial diversity according to Binetruy *et al*. (31). First, the tick samples were placed in a sterile disposable Petri dish and washed twice for 3 minutes each using 1% NaClO. They were then washed three times with pure water for 2 minutes each. After air-drying, they were placed in 2-mL sterile centrifuge tubes with 50 mL of PBS solution added. The ticks were then pulverized using a hand-held homogenizer. Following these steps, DNA extraction was performed using the QIAmp DNA Investigator kit (56504). The purity and concentration of DNA were then detected using 1% agarose gel electrophoresis and NanoDrop 2000 spectrophotometry (Thermo, USA).

### Amplification, quantification, library construction, and sequencing

Qualified DNA samples were transported on dry ice to Novogene Co., Ltd. Subsequent library construction, 16 s rRNA sequencing, and metagenomic sequencing were carried out in strict accordance with the company’s protocols. Specific methods: 16S V3–V4 primers (341F: CCTACGGRBGCACAG; 806R: GGACTACNNGGTATCTAAT) were selected to identify bacterial diversity. All PCRs were carried out with 15 µL of Phusion High-Fidelity PCR Master Mix (New England Biolabs), 0.2 µM of forward and reverse primers, and about 10 ng of template DNA. Thermal cycling consisted of initial denaturation at 98℃ for 1 minute; followed by 30 cycles of denaturation at 98℃ for 10 seconds, annealing at 50℃ for 30 seconds, and elongation at 72℃ for 30 seconds, with a final elongation at 72℃ for 5 minutes. An equal volume of 1X loading buffer (contained SYB green) was mixed with PCR products and operated electrophoresis on 2% agarose gel for detection. PCR products were mixed in equimolar ratios and then purified. Sequencing libraries were generated, and indexes were added. The library was quantified using Qubit and real-time PCR, and size distribution was assessed using a bioanalyzer. Quantified libraries were pooled and sequenced on Illumina platforms based on the effective library concentration and the required data amount.

The metagenomic DNA was randomly sheared into short fragments and sequencing libraries. The obtained fragments were end-repaired, A-tailed, and further ligated with the Illumina adapter. The fragments with adapters were PCR-amplified, size-selected, and purified. The library was quantified with Qubit and real-time PCR for quantification, and the size distribution was assessed by a bioanalyzer. Quantified libraries were pooled and sequenced on Illumina platforms based on the effective library concentration and the required data amount.

### Sequence data analysis

Paired-end reads were assigned to samples based on their unique barcode and truncated by cutting off the barcode and primer sequence. Paired-end reads were merged using FLASH (V1.2.1 1, http://ccb.jhu.edu/software/FLASH/)(Magoc et al., 2011), a very fast and accurate analysis tool designed to merge paired-end reads when there is an overlap between the reads generated from the opposite ends of the same DNA fragment, and the splicing sequences were called raw tags. Quality filtering of the raw tags was performed using the fastp (Version 0 0.23. 1) software to obtain high-quality clean tags (32). The tags were compared with the reference database (Silva database (16S/18S), https://www.arb-silva.de/; Unite Database (ITS), https://unite.ut.ee/) using the UCHIME algorithm (http://www.drive5.com/usearch/manual/uchime_algo.html) to detect chimera sequences, which were then removed (33). The effective tags were finally obtained. Denoising of effective tags was performed using the DADA2 or deblur module in the QIIME2 software (Version QIIME2-202202) to obtain initial amplicon sequence variants (ASVs), with DADA2 as the default. Species annotation was then performed using QIIME2 software. The raw reads were deposited into the NCBI Sequence Read Archive (SRA) database (BioProject ID: PRJNA1131649).

Readfq (https://github.com/cjfields/readfq) was used to preprocess raw data from the Illumina sequencing platform to obtain clean data for subsequent analysis. MEGAHIT software was utilized for assembly analysis of clean data. With the default parameters, MetaGeneMark (http://topaz.gatech.edu/GeneMark/) performs ORF prediction for Scaftigs (≥ 500 bp) in each sample. CD-HIT software (http://www.bioinformatics.org/cd-hit/) was then used to eliminate redundancy in ORF prediction results (34, 35) to obtain the nonredundant initial gene catalogue. The clean data of each sample are aligned to the initial gene catalog using Bowtie2, which calculates the number of reads for each gene in every sample alignment. Genes with reads ≤2 in each sample were filtered out to finalize the gene catalog (Unigenes) for subsequent analysis. DIAMOND software (https://github.com/bbuchfink/diamond/) was used to align Unigene sequences with NCBI’s NR database and KEGG database (http://www.kegg.jp/kegg/) for annotations on species and functions (36). Unigenes were aligned to the CARD database (https://card.mcmaster.ca/) using the Resistance Gene Identifier (RGI) software (37) provided by the CARD database (38). According to the RGI alignment result and unigene abundance information, the relative abundance of each ARO is calculated. The raw reads were deposited into the NCBI Sequence Read Archive (SRA) database (BioProject ID: PRJNA1132781).

### Data processing and statistical analysis

Using the 16S rRNA sequencing results, we separately analyzed the alpha and beta diversity of the microbiomes for male ticks collected in summer (M1 group), female ticks collected in summer (F1 group), male ticks collected in autumn (M2 group), and female ticks collected in autumn (F2 group) (Table 1). These analyses were conducted using the vegan package in R (version 4.2.3). Shannon exponential box plots were generated using the ggviolin package, and the Wilcoxon tests between every two groups were performed with the ggpubr package. Plots of principal coordinate analysis (PCoA) based on the Bray–Curtis distance were plotted using the ggplot2 package. The difference between every two groups was performed using permutational multivariate analysis of variance (Adonis).

Metagenomic sequencing data were initially species-annotated. Based on the species annotation results of metagenomic sequencing, the abundance proportions of each phylum, genus, and species were calculated. The top 30 microorganisms in terms of relative abundance at both the genus and species levels, as well as the unannotated microorganisms grouped as “Others,” were displayed in a heatmap using the pheatmap package. The linear discriminant analysis effect size (LEfSe) tool was then employed to identify significant differential bacteria on species level in subgroups based on linear discriminant analysis (LDA) values (LDA >3.0). The distribution and mixed infection characteristics of potentially pathogenic symbionts according to 16S rRNA sequencing data were mapped through the UpSetR package in R (4.2.3), based on the potentially pathogenic symbionts identified at the species level by metagenomic sequencing. The KEGG pathway annotation results were plotted as histograms using the ggplot2 package. The obtained ARGs were subjected to Spearman correlation analysis (*P* < 0.05, |r| > 0.4) with the three potentially pathogenic symbionts in R (4.2.3) using the psych package and presented as a covariance network plot on Gephi (0.10.1). The LDA tool was then employed to identify significant ARGs in subgroups based on values (LDA >4.0). In this study, differences were deemed significant when *P* < 0.05 and extremely significant when *P* < 0.01.

## RESULTS

### The bacterial diversity in ticks

**Fig 2:**
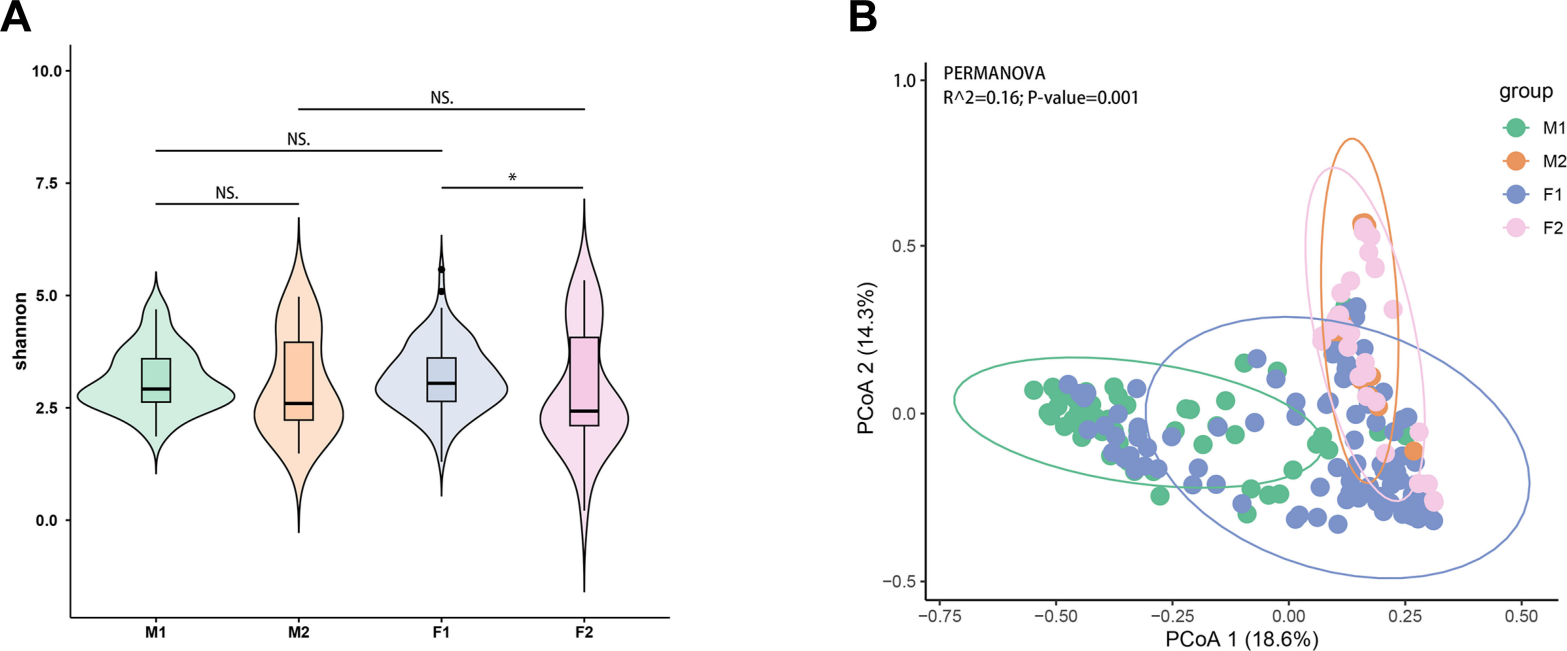
The differences in the bacterial diversity of male and female ticks collected in summer and fall. (A) The Shannon index of the microbiomes in different groups. (B) The PCoA result of the microbiomes in different groups. X-axis, PCoA axis 1 and Y-axis, PCoA axis 2. The scales of the X-axis and the Y-axis are the projection coordinates of the sample points in the two-dimensional plane. A dot represents each sample, and different colors represent different groups. M1: Male ticks collected in summer; M2: male ticks collected in fall; F1: female ticks collected in summer; F2: female ticks collected in fall; NS. *P* > 0.05; *P < 0.05.

Our result showed that there is no significant difference in the microbial diversity between ticks of different sexes in the same season (M1 vs F1, *P* = 0.536; M2 vs F2, *P* = 0.681, Fig. 2A). The diversity of the F1 group was significantly higher than that in F2 (*P* = 0.027), indicating that microbial diversity in female ticks is strongly influenced by season and enriched in summer (Fig. 2A). However, there was no significant difference in the alpha diversity of microbial communities in male ticks collected in different seasons (*P* = 0.343, Fig. 2A).

### The bacterial community structure in ticks

The PCoA analysis based on Bray–Curtis distances revealed community structure characteristics among the M1, M2, F1, and F2 groups. The M1 group exhibited higher dispersion among samples and showed clear separation in structure compared to M2 and F2 groups. The microbial communities of the M2 group partially overlapped with those of the F2 group, indicating a higher similarity in microbial composition among ticks collected in the same season. The first two components accounted for 18.6% and 14.3% of the total variation (Fig. 2B). Statistical verification using Adonis confirmed significant separation of the four groups (Adonis, R^2^ = 0.16, *P* = 0.001, Fig. 2B). Pairwise comparisons between groups showed there were highly significant differences in beta diversity between M1 and M2 (Adonis, R^2^ = 0.186, *P* = 0.001), M1 and F1 (Adonis, R^2^ = 0.097, *P* = 0.001), and F1 and F2 (Adonis, R^2^ = 0.070, *P* = 0.001, Fig. 2B), while there was no significant difference in beta diversity between female and male ticks collected in the fall (Adonis, R^2^ = 0.023, *P* = 0.379, Fig. 2B). Our results indicated that the microbial community structure carried by ticks is more significantly influenced by seasonal changes than by sex.

### The bacterial composition differences in ticks

A total of 45,298 ASVs were identified in 210 samples, annotated to 63 phyla. The dominant phylum of the four groups of ticks was the same, consisting mainly of Proteobacteria (76.12%), Firmicutes (14.97%), Bacteroidetes (3.89%), Actinobacteriota (2.00%), Cyanobacteria (0.51%), Desulfobacterota (2.70%), Fusobacteriota (0.22%), Deferribacterota (0.13%), Verrucomicrobiota (0.13%), and Acidobacteriota (0.12%). Male and female ticks collected in the same season exhibited similar phylum compositions.

**Fig 3:**
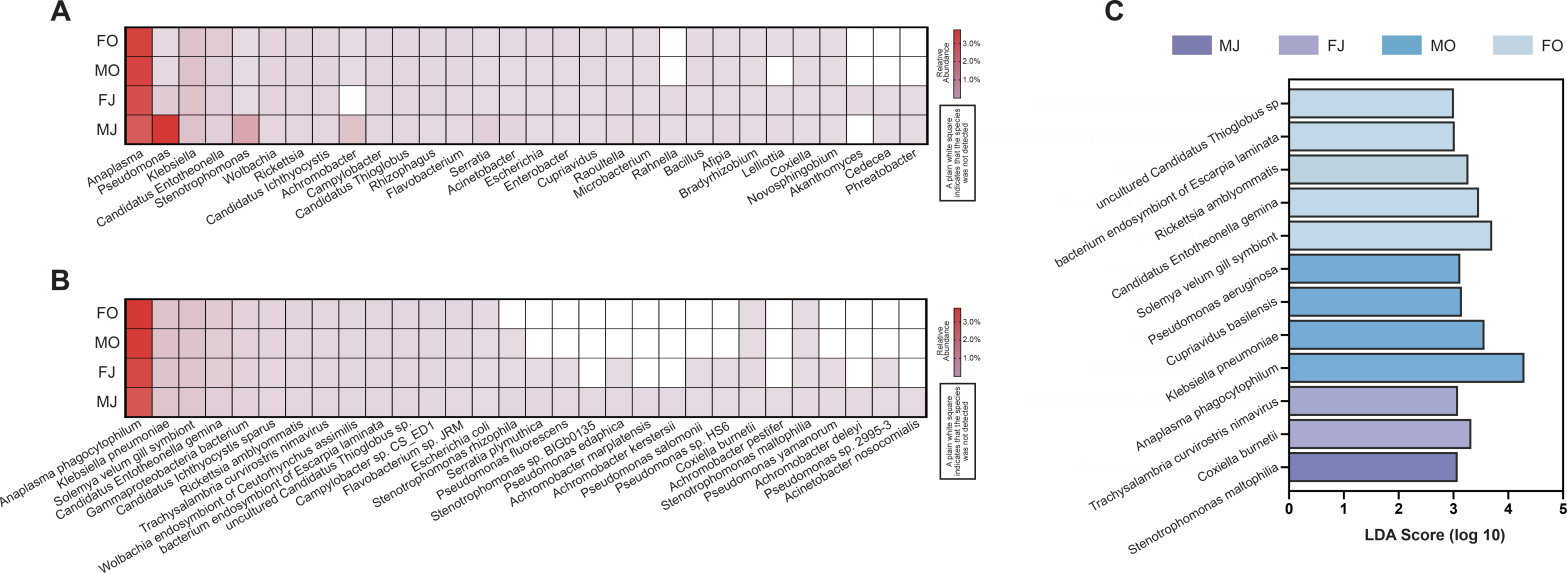
The differences in the composition of the microbiomes of male and female ticks collected in summer and fall. (A) The relative abundance of top 30 phyla in different groups. (B) The relative abundance of top 30 genera in different groups. (C) The linear discriminant analysis (LDA) demonstrated distinct microorganisms on each level enriched in different groups. When the default LDA value is >3.0 and the *P* < 0.05, the result corresponds to a differential species. MJ: Male ticks collected in summer; MO: male ticks collected in fall; FJ: female ticks collected in summer; FO: female ticks collected in fall.

Through metagenomic analysis, a total of 1,560 genera were identified across all samples, with the highest relative abundance noted for *Anaplasma* (3.46%), followed by *Pseudomonas* (1.10%), *Klebsiella* (0.75%), *Candidatus Entotheonella* (0.41%), *Stenotrophomonas* (0.39%), *Wolbachia* (0.21%), *Rickettsia* (0.20%), *Candidatus Ichthyocystis* (0.19%), *Achromobacter* (0.18%), and *Campylobacter* (0.13%, Fig. 3A). The top 30 genera in relative abundance accounted for approximately 7.86% of all genera, with their relative abundance ranging from 0 to 3.75% (Fig. 3A). By the same method, we identified a total of 4,336 bacterial species across all tick samples. The top 10 bacterial species in relative abundance exhibited a similar distribution across four groups. The average relative abundances of the top 10 species were as follows: *Anaplasma phagocytophilum* (3.46%), *Klebsiella pneumoniae* (0.73%), *Solemya velum gill symbiont* (0.70%), *Candidatus Entotheonella gemina* (0.41%), *Gammaproteobacteria bacterium* (0.27%), *Candidatus Ichthyocystis sparus* (0.20%), *Rickettsia amblyommatis* (0.19%), *Trachysalambria curvirostris nimavirus* (0.18%), *Wolbachia endosymbiont of Ceutorhynchus assimilis* (0.15), and *bacterium endosymbiont of Escarpia laminate* (0.13%). In addition, we found that 13 of the top 30 relative abundance bacterial species were not detected in ticks during the fall, suggesting that the dominant bacterial species are more diverse in ticks during the summer (Fig. 3B).

Through LDA, we further discovered that *Stenotrophomonas maltophilia* (LDA Score = 3.078, *P* = 0.043) is enriched in male ticks during the summer, while *Coxiella burnetii* (LDA Score = 3.327, *P* = 0.003) and *Trachysalambria curvirostris nimavirus* (LDA Score = 3.082, *P* = 0.012) are enriched in female ticks during the same season (Fig. 3C). In the fall, *Anaplasma phagocytophilum* (LDA Score = 4.294, *P* = 0.015), *Klebsiella pneumoniae* (LDA Score = 3.568, *P* = 0.009), *Cupriavidus basilensis* (LDA Score = 3.153, *P* = 0.002), and *Pseudomonas aeruginosa* (LDA Score = 3.124, *P* < 0.001) are enriched in male ticks, whereas *Solemya velum gill symbiont* (LDA Score = 3.707, *P* = 0.001), *Candidatus Entotheonella gemina* (LDA Score = 3.465, *P* = 0.003), *Rickettsia amblyommatis* (LDA Score = 3.273, *P* = 0.002), *bacterium endosymbiont of Escarpia laminate* (LDA Score = 3.023, *P* = 0.007), and *uncultured Candidatus Thioglobus sp*. (LDA Score = 3.010, *P* = 0.029) are enriched in female ticks (Fig. 3C). Understanding the sex-specific and seasonal distribution of these bacterial species can aid in the targeted prevention and management of tick-borne bacterial diseases in giant pandas.

### Analysis of potentially pathogenic symbionts and mixed infections

Based on metagenomic species composition analysis, we identified three tick-borne common potentially pathogenic symbionts: *Anaplasma phagocytophilum*, *Coxiella burnetii*, and *Rickettsia amblyommatis*. These symbionts were found with a 100% infection rate in all 36 tick samples. To further investigate the widespread infection rates of these potentially pathogenic symbionts in *I. ovatus*, we conducted a co-infection analysis of the corresponding genera using 16S rRNA data. By 16S rRNA genera identification, *Coxiella*, *Rickettsia,* and *Anaplasma* were found in the microbial community of all tick samples, with carriage rates of 90.00%, 13.81%, and 8.10%, respectively. Out of 210 tick samples, only 21 ticks were free of these potentially pathogenic symbionts. Among the other 189 ticks, 82.54% were infected with a single potentially pathogenic symbiont (*Coxiella*), while 10.58% and 6.88% had compound infection with two and three potentially pathogenic symbionts, respectively. There were two combinations of compound infection involving two potentially pathogenic symbionts, all of which included Coxie*lla* with one of the other two potentially pathogenic symbionts (*Coxiella* and *Rickettsia*: 8.46%, *Coxiella* and *Anaplasma*: 2.12%). Similarly, there was only one combination of compound infection involving three potentially pathogenic symbionts, also including *Coxiella* (6.88%).

### Annotation of microbial gene function

**Fig 4:**
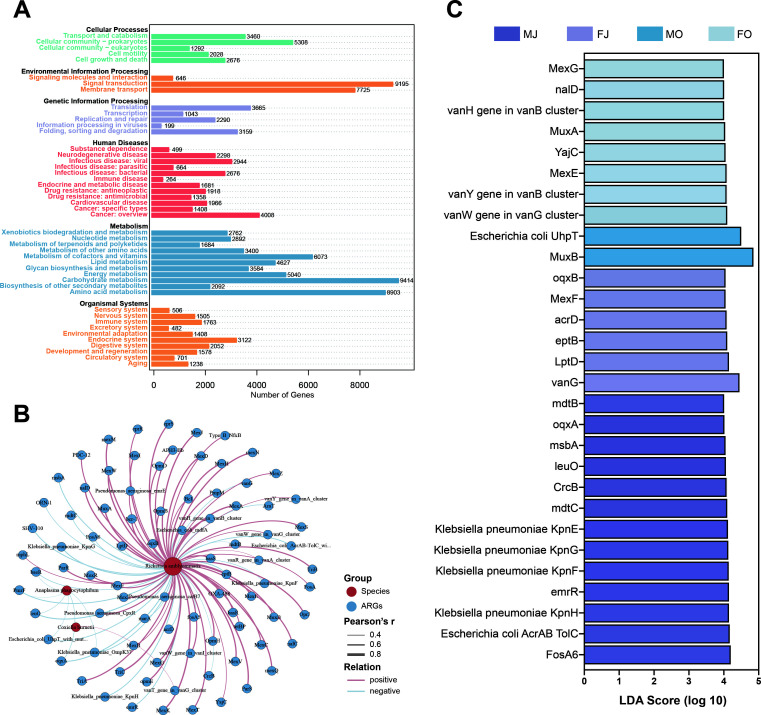
Microbial gene function annotation diagram and antibiotic resistance gene analysis. (A) KEGG pathway annotation bar chart; (B) Network analysis on antibiotic resistance genes with three potentially pathogenic symbionts (*Anaplasma phagocytophilum*, *Coxiella burnetii,* and *Rickettsia amblyommatis*). ARGs: antibiotic resistance genes; species: strain; positive: positive correlation; negative: negative correlation. (C) The linear discriminant analysis (LDA) demonstrated distinct ARGs enriched in different groups. When the default LDA value is >4.0 and the *P* < 0.05, the result corresponds to a differential species. MJ: Male ticks collected in summer; MO: male ticks collected in fall; FJ: female ticks collected in summer; FO: female ticks collected in fall.

The gene annotation information was obtained by comparing the sequences of nonredundant gene sets with the KEGG gene database. A total of 46 pathways were identified in the microorganisms carried by ticks, with the most pathways related to human diseases (12/46, 26.09%), followed by metabolism (11/46, 23.91%), organismal systems (10/46, 21.74%), cellular processes (5/46, 10.87%), genetic information processing (5/46, 10.87%), and environmental information processing (3/46, 6.52%). The pathways associated with human diseases were related to substance dependence, neurodegenerative diseases, viral infectious diseases, parasitic infectious diseases, bacterial infectious diseases, immune diseases, endocrine and metabolic diseases, antineoplastic resistance, antimicrobial resistance, cardiovascular diseases, specific types of cancers, and other overview cancers (Fig. 4A). It provides important information for the in-depth exploration of the relationship between tick-borne microorganisms and human diseases and provides a developmental direction for future research.

Comparison with the comprehensive antibiotic resistance database (CARD) resulted in a total of 121 antibiotic resistance genes, and the analysis of different gene resistance mechanisms revealed 76 ARGs. Similarly, we attempted to identify ARGs associated with the three tick-borne potentially infectious symbionts (*Anaplasma phagocytophilum*, *Coxiella burnetii*, and *Rickettsia amblyommatis*) of interest using Pearson correlation analysis. The results showed that the three species were correlated with 85 ARGs (|r| > 0.4, *P* < 0.05), and 92 linkages were obtained, with positive correlations (64.13%) being much higher than that of negative (35.87%, Fig. 4B). *Rickettsia amblyommatis* was negatively correlated with 27 ARGs and positively correlated with 58 ARGs, which was considered a multidrug-resistant bacterium (Fig. 4B). *Anaplasma phagocytophilum* was negatively correlated with six ARGs, which were linked to the use of aminoglycoside antibiotics, peptide antibiotics, and phosphonic acid antibiotics (Fig. 4B). *Coxiella burnetii* was positively correlated only with the vanT gene in the vanG cluster, which was resistant to glycopeptide antibiotics (Fig. 4B).

In addition to conducting correlation analyses of ARGs associated with specific potentially pathogenic symbionts, we used LDA to compare and select ARG markers across different sex and seasons. We found that male ticks in summer had the highest enrichment of ARGs, with 13 ARGs including mdtB, oqxA, and msbA (Fig. 4C). For female ticks in summer, six ARGs were enriched, including oqxB, MexF, and acrD (Fig. 4C). In female ticks during autumn, eight ARGs were enriched, such as MexG, nalD, and MuxA (Fig. 4C). The least enrichment was observed in male ticks in autumn, with only two ARGs significantly enriched: MexE and *Escherichia coli* UphT (Fig. 4C).

## DISCUSSION

Our previous research on the infection factors, taxonomy, and virology of *I. ovatus* in this region served as a basis for the current study (14, 15, 25, 39). Using morphological methods, all 210 ticks were identified as *I. ovatus. I. ovatus* could infect giant pandas, causing babesiosis with symptoms like anemia, lethargy, and rapid weight loss (14). Additionally, pathogens it carries, such as *Rickettsia*, may lead to Japanese spotted fever, threatening the health of giant pandas and other species (19). Model predictions suggest that future climate change will promote the expansion of *I. ovatus* in distribution and population across China (40). This highlights the necessity of strengthening research on its potential pathogenic symbionts for the conservation of giant panda populations. In this study, we analyzed the microbiota’s sexual and seasonal structural and diversity changes in giant panda-infesting ticks (*I. ovatus*) via 16S rRNA sequencing, accurately explored their composition and identified potentially pathogenic symbiont species through metagenomic sequencing, and recommended potential therapeutic antibiotics based on ARG enrichment characteristics.

Studies have shown that female ticks of the same species typically carry lower bacterial diversity than male ticks within the same habitat (41). This may be because sex could indirectly influence the interactions between ticks and microorganisms by affecting the host’s immune system and metabolic activities (42). For example, there are significant differences in how male and female *Aedes albopictus* respond to the pathogen *Mycobacterium avium subsp. paratuberculosis* and the probiotic *Lactobacillus animalis NP-51* (43). In the study of *Ixodes ovatus*, *Ixodes persulcatus*, and *Amblyomma variegatum*, there are significant differences in the gene abundance of metabolic pathways such as vitamin B, amino acids, carbohydrates, nucleotides, and antibiotics between males and females, reflecting how sex-related physiological characteristics significantly affect their interactions with the microbiome (44). Our result showed that no statistically significant differences in microbial diversity were observed between male and female *I. ovatus* in either summer or autumn. Instead, seasonal changes had a more substantial impact on the microbial diversity of *I. ovatus*, with female individuals exhibiting higher microbial diversity in the summer. Perveen *et al*. showed that the abundance of tick microbial communities differed between months, with the bacterial community in the summer months (June and July) forming one cluster and the rest of the months forming another (24). We speculate that, on one hand, seasonal changes in the temperature and humidity may affect the activity patterns and life cycles of ticks, thereby influencing their interactions with microorganisms (45). On the other hand, from the perspective of the tick’s host, the host’s physiological state, and microbial communities and composition, even the immune system can be impacted by seasonal changes (46). As part of the host, the microbial communities within ticks are influenced by the host and may also undergo similar seasonal variations.

Our results showed that the dominant phyla in ticks mainly consisted of Proteobacteria and Firmicutes, which was consistent with the results of Xiang *et al*. (47). Based on metagenomic techniques, we identified that the primary bacterial genus carried by *I. ovatus* ticks in the Daxiangling region is *Anaplasma*, consistent with the findings of previous studies (48). The genera *Pseudomonas* and *Klebsiella* have been identified in *Ixodes granulatus*, *Haemaphysalis wellingtoni*, *Haemaphysalis hystricis*, and *Haemaphysalis bispinosa* before (49, 50). This study is the first to report *Pseudomonas* and *Klebsiella* as dominant genera in *I. ovatus*. From the perspective of investigating and controlling tick-borne diseases, this study primarily focuses on bacterial symbionts with potential pathogenic capabilities in ticks. Through metagenomic species identification, and by referencing commonly recorded tick-borne pathogens in the literature, our study primarily targeted three bacterial species: *Anaplasma phagocytophilum*, *Coxiella burnetii*, and *Rickettsia amblyommatis. A. phagocytophilum* is a gram-negative bacterium transmitted by ticks of the *I. ovatus*, and it is widely distributed across the globe, including in Europe, the United States, and Asia (51, 52). In the United States, the infection rate of *A. phagocytophilum* in *Ixodes scapularis* ranges from 1% to 50% (52). In Europe, the infection rate of *A. phagocytophilum* in *Ixodes ricinus* ranges from 1% to 20% (52). In our study, metagenomic analysis of 36 *I. ovatus* samples revealed a 100% infection rate of *A. phagocytophilum*, and the overall infection rate of the genus *Anaplasma* in *I. ovatus* was 8.1%, which indicates a significant potential pathogenic risk associated with *A. phagocytophilum. A. phagocytophilum* can infect both myeloid and non-myeloid cells of vertebrate hosts through tick bites, particularly targeting neutrophils (52–54). Within host cells, *A. phagocytophilum* replicates by forming parasitic vacuoles, allowing it to evade the host cell’s defense mechanisms (52, 54). *A. phagocytophilum* can cause weight loss, fever, abortion, infertility, and even death in wild boars (*Sus scrofa*), sika deer (*Cervus nippon*), and squirrels (*Sciurus vulgaris*) (53, 55, 56). Considering that these animals share similar habitats with the giant panda, it is important to pay attention to the potential impact of *A. phagocytophilum* on giant pandas. *I. ovatus* serves as an important vector for the transmission of *C. burnetii* between wild vertebrates and livestock (57, 58). *C. burnetii* is widely distributed globally, encompassing almost the entire world, including the Arctic (59, 60). Animals’ *C. burnetii* infection (Q fever) can manifest as an acute disease, typically presenting as a self-limiting flu-like illness, pneumonia, or hepatitis, or as a chronic form, primarily characterized by endocarditis, but also including hepatitis and chronic fatigue syndrome (61). The reported prevalence of *C. burnetii* infection in bears varies across different regions, with rates of 78% in black bears (*Ursus thibetanus*) from eight prefectures in Japan, 8% in black bears (*Ursus Americanus Floridanus*) from Florida, USA, and 9% in brown bears (*Ursus arctos*) from Croatia (17, 62, 63). These findings highlight the exposure risk of giant pandas to *C. burnetii* infection, given its occurrence in bear species across different continents. The infection rate of *Rickettsia* in *I. ovatus* is 13.26%, primarily involving *Rickettsia asiatica* (19, 64). This study is the first to clearly identify that *I. ovatus* can also be infected with *R. amblyommatis*. Researchers have detected *R. amblyommatis* in *Amblyomma americanum* and *Dermacentor variabilis* carried by black bears (*Ursus americanus amblyceps*), with detection rates of 60% and 35%, respectively (65). Infection with *R. amblyommatis* in wildlife can lead to weight loss, antibody suppression, and reproductive system changes (66). In Rhesus monkeys (*Macaca mulatta*), severe *R. amblyommatis* infection can alter fluid and electrolyte distribution, adversely affecting the host’s long-term health (67). There is also evidence suggesting that *R. amblyommatis* can elicit an immune response in certain hosts, providing protection against other more pathogenic *Rickettsia* spp. (68). For instance, in a guinea pig (*Cavia porcellus*) model, animals exposed to *R. amblyommatis* remained healthy when subsequently challenged with a lethal dose of *Rickettsia rickettsii*, whereas non-immunized controls developed severe disease and died (68). As a symbiont in ticks infesting giant pandas, the pathogenic potential of *R. amblyommatis* to giant pandas warrants particular attention.

Meanwhile, our study identified conditionally pathogenic bacteria such as *Klebsiella pneumoniae* and *Pseudomonas aeruginosa* (69, 70). Recent studies have indicated that infection with *K. pneumoniae* in giant pandas primarily manifests in two pathological forms: enteritis and septicemia (70, 71). The enteritis form is characterized by symptoms such as lethargy, reduced appetite, emaciation, and diarrhea, whereas the septicemia form may lead to systemic infection, affecting multiple organs and potentially resulting in death (71). The transmission risk of *K. pneumoniae* among giant pandas is relatively high, and the bacterium exhibits significant antibiotic resistance. The presence of multidrug-resistant *K. pneumoniae* (*MDR K. pneumonia*) strains isolated from panda feces further complicates treatment efforts (69). Giant pandas infected with *P. aeruginosa* mainly suffer from diarrhea and respiratory infections (72). A case report has documented that giant pandas got pneumonia due to *P. aeruginosa* infection and subsequently lead to secondary enteritis, indicating that *P. aeruginosa* can pose serious health threats to giant pandas (73). Our study provided evidence of the presence of *K. pneumoniae* and *P. aeruginosa* in ticks infesting giant pandas, which may aid in the rapid diagnosis of diseases in the future. Besides, our LDA indicates that in the Daxiangling region of China, efforts should focus on preventing *C. burnetii* infections during the summer, while in the autumn, the focus should shift to preventing infections by *A. phagocytophilum*, *K. pneumoniae*, *P. aeruginosa*, and *R. amblyommatis*. Effective strategies for the prevention and control of tick-borne diseases should be formulated based on the species of ticks and the seasonal variation of the pathogens they carry (24, 45).

Multi-pathogen co-infection has been found in a variety of tick species in previous studies, such as the *Ixodes scapularis* in the northeastern United States, the *Ixodes pacifica* and *Ixodes spinipalpus* in the western United States, the *Ixodes ricinus* in Europe, and the *Ixodes persuleatus* in Asia (74). Bites from ticks carrying multiple pathogens increase the risk of simultaneous infection of the host with multiple pathogens, leading to variability and worsening of clinical symptoms (74). Reports have documented cases where giant pandas succumbed to systemic multi-organ dysfunction caused by dual infections of *K. pneumoniae* and *Proteus mirabilis* following trauma (75). Additionally, mixed infections involving *Escherichia coli* and *K. pneumoniae* have been implicated in the death of a subadult giant panda (76). Mixed infections accelerate disease progression and complicate management, with the resulting multidrug resistance further exacerbating treatment challenges (77). In the Al Dhafra region of Abu Dhabi, the co-infection rate of *C. burnetii* and *A. phagocytophilum* in camels (*Camelus dromedarius*) is 1.1%, resulting in variable acute clinical signs, including pyrexia, anorexia, swelling of external lymph nodes, edematous swellings, and lacrimation (78). Ticks collected in France exhibit a co-infection rate of 5.1% with *A. phagocytophilum* and *Rickettsia* spp., which have been associated with a significant increase in the spring mortality rate of Pyrenean chamois (*Rupicapra pyrenaica pyrenaica*) in the region (79). Additionally, *Ixodes ricinus* and *Ixodes inopinatus* ticks collected from roe deer (*Capreolus capreolus*), wild boar (*Sus scrofa*), red deer (*Cervus elaphus*), brown bears (*Ursus arctos*), badgers (*Meles meles*), and foxes (*Vulpes vulpes*) in western and middle Slovakia have been found to harbor multiple infections with *A. phagocytophilum*, *C. burnetii*, and *Rickettsia* spp. (80). This highlights the significance of the *Ixodes* sp. as a host for *A. phagocytophilum*, *C. burnetii*, and *Rickettsia* spp., as well as the importance of these co-infections as a threat to wildlife health (80). The co-infection rate of the three potentially pathogenic symbionts (*Anaplasma*, *Coxiella* and *Rickettsia*) in this study is 6.88%, which is significantly higher than the 0.92% infection rate observed in the Heilongjiang border region of China (81). This study is the first to identify co-infections of *A. phagocytophilum*, *C. burnetii,* and *R. amblyommatis* in *I. ovatus* ticks infesting giant pandas, underscoring the risk of tick-borne diseases that giant pandas face in their natural habitats.

Wild animals are considered reservoirs of ARGs (82). Analyzing the correlation between microbial ARGs and strains in ticks enhances our understanding of the pathogenesis of relevant bacteria and their interactions with the host, guiding clinical medication and improving the therapeutic efficacy (69, 70). In terms of treating infections caused by a single potentially pathogenic symbiont, our correlation analysis revealed that *R. amblyommatis* exhibits characteristics of multidrug resistance. The 27 significantly negatively correlated ARGs are primarily associated with mechanisms of antibiotic efflux and changes in membrane permeability. Based on related studies, it is suggested that tetracycline and β-lactam antibiotics be used for treatment (83). Using the same methodology, we recommend the use of aminoglycoside antibiotics, peptide antibiotics, and phosphonic acid antibiotics for the treatment of *A. phagocytophilum* infections and use of glycopeptide antibiotics to treat *C. burnetii* infections. In cases of mixed infections with multiple unknown pathogens, metagenomic capture and enrichment techniques can more accurately identify resistance elements and play a crucial role in the diagnosis and treatment of bacterial infections (84). Based on the LDA results, we recommend avoiding the use of antibiotics associated with efflux mechanisms and changes in membrane permeability, such as chloramphenicol, certain β-lactam antibiotics, and fluoroquinolones, when treating tick-borne diseases in the summer. For treating tick-borne diseases in the autumn, we suggest avoiding antibiotics related to efflux pump genes and those involved in metabolism and transport, such as fosfomycin, aminoglycosides, and macrolides.

This study is the first to identify the sexual and seasonal differences in the microbial community structure of ticks infecting giant pandas. It also identifies potential pathogenic symbionts and explores their antibiotic resistance genes, providing a basis for the prevention and control of tick-borne diseases. Future research could focus on the following areas: 1) expand sampling strategies: broaden the geographic and individual coverage of giant pandas, integrate multi-source data, and build precise predictive models to enhance risk assessment and control capabilities for tick-borne diseases. 2) Deepen mechanistic studies: utilize multi-omics technologies along with cell biology and animal models to analyze infection pathways, host immune responses, and disease progression, providing targets for prevention and control. 3) Strengthen integration of environmental factors: include environmental factor monitoring to construct comprehensive models that quantify the environment–tick–pathogen–host interaction network, offering strategies for tick-borne disease prevention under changing ecosystem conditions.

## Data Availability

The data sets generated during the current study are available in the NCBI repository. The 16S rRNA sequencing data are available at BioProject accession no. PRJNA1131649. The metagenomic sequencing data are available at BioProject accession no. PRJNA1132781
